# Kisspeptin and Testicular Function—Is It Necessary?

**DOI:** 10.3390/ijms21082958

**Published:** 2020-04-22

**Authors:** Aditi Sharma, Thilipan Thaventhiran, Suks Minhas, Waljit S. Dhillo, Channa N. Jayasena

**Affiliations:** 1 Section of Investigative Medicine, Imperial College, 6th Floor, Commonwealth Building, Hammersmith Hospital, 150 Du Cane Road, London W12 0NN, UK; aditi.sharma@imperial.ac.uk (A.S.); thilipan.thaventhiran@nhs.net (T.T.); w.dhillo@imperial.ac.uk (W.S.D.); 2 Department of Urology, Imperial College Healthcare NHS Trust, Charing Cross Hospital, Fulham Palace Road, Hammersmith, London W6 8RF, UK; suks.minhas@nhs.net

**Keywords:** kisspeptin, kisspeptin receptor, spermatozoa, Leydig cells, Sertoli cells, testes, testosterone, LH, FSH, spermatogenesis

## Abstract

The role of kisspeptin in stimulating hypothalamic GnRH is undisputed. However, the role of kisspeptin signaling in testicular function is less clear. The testes are essential for male reproduction through their functions of spermatogenesis and steroidogenesis. Our review focused on the current literature investigating the distribution, regulation and effects of kisspeptin and its receptor (KISS1/KISS1R) within the testes of species studied to date. There is substantial evidence of localised KISS1/KISS1R expression and peptide distribution in the testes. However, variability is observed in the testicular cell types expressing KISS1/KISS1R. Evidence is presented for modulation of steroidogenesis and sperm function by kisspeptin signaling. However, the physiological importance of such effects, and whether these are paracrine or endocrine manifestations, remain unclear.

## 1. Introduction

Kisspeptin is an established regulator of puberty onset [1,2], sexual maturation and adult reproductive activity [3]. Several studies confirm that kisspeptin acts on KISS1R on hypothalamic gonadotrophin-releasing hormone (GnRH) neurons, which in turn stimulate gonadotrophins (luteinising hormone (LH), follicle-stimulating hormone (FSH)) and downstream sex hormones (testosterone and oestradiol) [4,5]. The ability of kisspeptin to stimulate reproductive hormones was confirmed in man in 2005 by the first ‘kisspeptin into human’ study [6]. Disruption to the central KISS1/KISS1R system leads to various reported reproductive disorders. Inactivating mutations of KISS1R cause hypogonadotropic hypogonadism [7,8,9]; conversely, activating mutations cause precocious puberty [1,7,10]. The central role of kisspeptin in stimulating hypothalamic GnRH secretion is undisputed. Yet, the peripheral role of kisspeptin in tissues such as the testes is less well understood. The testes are essential for male reproduction through their two prime functions, namely spermatogenesis and steroidogenesis. Multiple studies have observed the distribution, expression and activity of both KISS1/KISS1R in the testes [11,12,13,14]. Furthermore, re-expression of KISS1R in GnRH neurons is insufficient to normalise testicular function, suggesting a potential direct role of KISS1/KISS1R in testis physiology [15]. Studies in primates [16,17,18], rodents [19,20,21], amphibians [22,23,24] and fish [25,26] have observed potential regulatory roles of kisspeptin in germ cell progression, modulation of sperm function and testicular steroidogenesis. In this review, we discuss evidence of local expression of KISS1/KISS1R system in the testes and its role in direct testicular functions. 

## 2. Testicular Function

Spermatogenesis is an intricate process whereby male germ cells (spermatogonia) develop into mature spermatozoa through the processes of mitosis, meiosis and cell differentiation. In men, spermatogenesis occurs in the recesses of the Sertoli cells located along the entire length of the seminiferous tubules (ST) of the testes [27]. Spermatogenesis is regulated by several cell types, hormones, genetic and epigenetic factors [28]. Sertoli cells nourish and provide structural support to germ cells, and Leydig cells synthesise steroid hormones essential for spermatogenesis. Mammalian spermatozoa undergo multiple steps to successfully fertilise the oocyte including maturation in the epididymis, capacitation and acrosome reaction in the female reproductive tract [29,30]. These testicular functions are dependent on the hypothalamic-pituitary-gonadal (HPG) axis [31]. Hypothalamic pulsatile secretion of GnRH stimulates LH and FSH from the anterior pituitary to act on the testes. FSH stimulates Sertoli cell function and spermatogenesis. Sertoli cells also produce inhibin B which is a marker of spermatogenesis. LH stimulates Leydig cells to synthesize testosterone. Testosterone and Inhibin B have negative feedback effects at the pituitary and hypothalamic levels. Optimal spermatogenesis requires the action of both testosterone and FSH. Derangements at any of these steps can cause male infertility [32].

## 3. Distribution of Kisspeptin and Its Receptor

**Figure 1:**
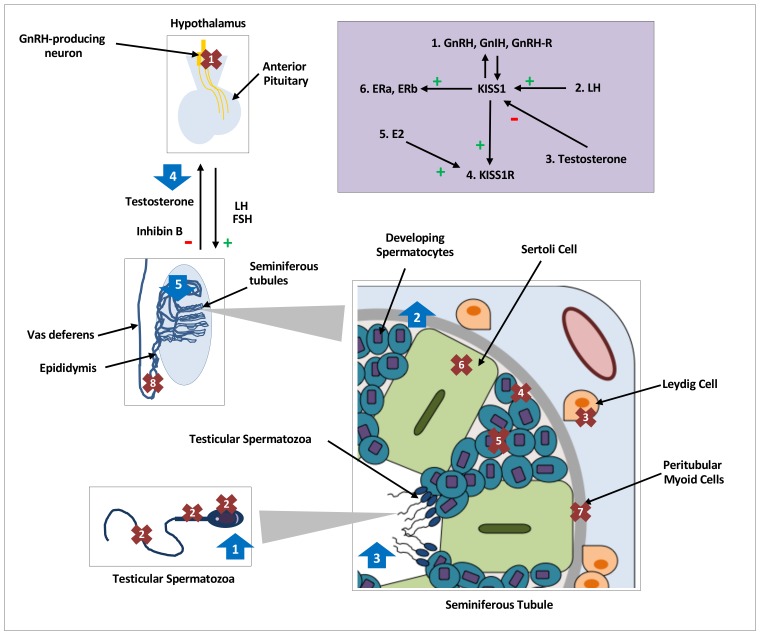
Distribution, regulation and effects of kisspeptin on testicular function in various species. Distribution and expression of testicular KISS1/KISS1R, denoted by crosses 1–8, regulation of testicular kisspeptin signaling, denoted by the purple shaded box and effects of kisspeptin on testicular function, denoted by arrows 1–5. GnRH, gonadotrophin releasing hormone; GnIH, gonadotrophin inhibiting hormone;, L.H.; luteinising hormone; FSH, follicle stimulating hormone; hCG, human chorionic gonadotropin; E2, oestradiol;, E.R.; oestrogen receptor; kp: kisspeptin. Crosses. 1: KISS1/KISS1R in central hypothalamic GnRH neurons; 2: KISS1/KISS1R in head, neck, and flagellum of human spermatozoa [16]; 3: KISS1 in Leydig cells of mice [19,20,21,42] and monkeys [18], and KISS1R in Leydig cells of mice [12,42]; 4: KISS1 in primordial germ cells of mice [19] and KISSR in proliferating germ cells of frogs [24]; 5: KISS1/KISS1R in developing spermatids and spermatocytes of mice [12,20], monkeys [17] and frogs [24]; 6: KISS1R in Sertoli cells of frogs [24] and monkeys [17,18]; 7: KISS1R in peritubular myoid cells of frogs [24]; 8: KISS1 in epididymal epithelium of mice [20]. Purple Box: 1: GnRH, GnIH and GnRH-R modulate KISS1 in an autocrine or paracrine manner [19]. 2: An increase in LH increases testicular KISS1 mRNA and protein [21]. 3: Testosterone decreases KISS1 expression and protein [21]. 4: kp induces expression of KISS1R [24]. 5: E2 induces expression of KISS1R [24]. 6: KISS1 induces expression of oestradiol receptors, ERa & ERb. Arrows. 1: Increased intracellular calcium in spermatozoa, increased sperm motility and hyperactivation in men [16]; 2: Increased spermatogonial cell proliferation and differentiation markers in adult mice [48]; 3: Increased number of seminiferous tubules with detached spermatozoa in frogs [43]; 4: Nil effect of kp on testosterone secretion with or without cotreatment with LH or hCG in mice [12,42]; 5. Continuous infusions of kp led to seminiferous tubule degeneration and apoptosis, and reduction in, L.H.; testosterone and inhibin B levels in mice [50,51].

Multiple isoforms of both KISS1 and KISS1R genes have been identified in non-mammalian vertebrates (fish and amphibians) whilst mammals only have one KISS1 and KISS1R gene [33,34,35,36]. Studies are summarised below into different species in order to highlight the inter- and intra-species differences (see Table 1 and Figure 1).

*Humans:* Pinto et al. [16] collected freshly ejaculated semen from fifty-six healthy normospermic donors and identified both KISS1 and KISS1R proteins in the head (post-acrosomal region), neck, and the flagellum of spermatozoa using Western blot and immunocytochemistry/immunofluorescence assays. In addition, Neurokinin B (NKB) immunoreactivity was co-localised at the head of sperm cells, particularly in the equatorial segment that is known to have an important role in oocyte–sperm fusion [37,38]. KNDy neurons (kisspeptin, NKB, opioid dynorphin A) are co-expressed in the hypothalamus with a coordinated role to modulate pulsatile GnRH and subsequent gonadotrophin secretion [39,40]. This was the first study to identify human spermatozoa as the first non-neuronal KNDy cell. This suggests possible co-expression of peripheral KNDy peptides in modulating local testicular GnRH action, however further studies to define this role are required. 

*Rodents:* Anjum et al. [19] evaluated the changes in the distribution and concentration of three closely related neuropeptides (GnRH; Gonadotrophin Inhibiting Hormone, GnIH and kisspeptin) in the mouse testis. They reported KISS1 expression using Western/slot blot analysis in Leydig cells of Parkes strain mice. In addition, KISS1 was identified in primordial and elongated germ cells of male mice. They did not report KISS1R expression in the male mouse testis. In contrast, Mei et al. [12] used β-galactosidase staining in transgenic mice with LacZ reporter gene inserted into the KISS1 and KISS1R alleles to detect gene expression. Both KISS1 and KISS1R mRNA were expressed by round spermatids beyond one month of the age of the mice. However, there was no KISS1 immunoreactivity in spermatids by immunohistochemistry when sections of testis were visualised. This may be secondary to multiple hypothetical reasons. Expression of kisspeptin protein levels may be below the level of detection by immunolocalization or translation of KISS1 gene may not occur till the late stages of spermiogenesis (beyond spermatid stage). Furthermore, the loss of cytoplasm during structural remodeling of spermatids to produce mature spermatozoa may have failed to demonstrate the cytoplasmic beta galactosidase staining post the spermatid stage. In addition, KISS1R expression was detected using reverse transcriptase-polymerase chain reaction (RT-PCR) in the Leydig MA-10 cell line; however, no detectable KISS1 expression was observed. Hsu et al. [20] differentiated KISS1 transcript levels in testicular interstitial cells and KISS1R transcript levels in the seminiferous tubules of adult mice by RT-PCR and immunoblot assay. Gene expression was confirmed by immunohistochemical assays. In contrast to Mei et al. [12], strong kisspeptin immunoreactivity was noted in both Leydig cells and in elongated spermatids; whereas, KISS1R was expressed in the acrosomal region of spermatids and mature spermatozoa but not in spermatogonial cells or spermatocytes of adult mice. KISS1R was observed in mice spermatozoa (similar to human spermatozoa [16]), and KISS1 was observed in Leydig cells, which have a major role in testicular steroidogenesis. These results suggest a possible paracrine signaling pathway between sperm and Leydig cells. Interestingly, KISS1 immunoreactivity was also found in the epididymis epithelium of adult mice. Epididymides and epididymal fluid provide an environment for spermatozoal storage and maturation [41]. Another study [21] quantified KISS1 mRNA expression as seventeen times higher in the mice testes than in hypothalamic arcuate nucleus (ARC). Similar to two earlier studies in mice [19,20], KISS1 protein expression was observed in Leydig cells by immunohistochemistry. KISS1R localisation was not reported. In contrast, Wang et al. [42] observed both KISS1 and KISS1R expression in adult mouse Leydig cells.

*Monkeys:* Studies in adult rhesus monkeys have supported the presence of KISS1 and KISS1R in the testes. Tariq et al. [17] reported KISS1 expression in spermatocytes and spermatids (i.e., immature sperm cells) versus the presence of KISS1R in spermatocytes and Sertoli cells, measured by dual immunocytochemistry and RT-PCR. Similar to rodents [19,20,21], Irfan et al. [18] reported KISS1 expression in Leydig cells of adult primates by immunocytochemistry. In accordance with the other study in adult monkeys [17] and to findings in amphibians [24], KISS1R was detected in Sertoli cells. The authors hypothesised the potential paracrine role of kisspeptin in Sertoli cells in influencing testosterone secretion by Leydig cells. Contrary to Tariq et al. [17], Irfan et al. [17] observed no immunostaining of germ cells for either KISS1 or KISS1R. 

*Frogs:* Chianese et al. [24] investigated KISS1R expression in the testes of this seasonal breeder. They observed KISS1R expression in the interstitial compartment and proliferating germ cells (primary spermatogonial, secondary spermatogonial and primary spermatocytes) of frog testes by in situ hybridization. They observed a seasonal fluctuation with increased mRNA expression in the testes and pituitary during breeding season (April) measured by RT-PCR. KISS1R mRNA was also detected in peritubular myoid cells suggestive of a potential local role of kisspeptin in sperm release and transport. There was no KISS1R expression in mature spermatozoa in contrast to the results of Pinto et al. [16] in men and Hsu et al. [20] in mice. No information was provided by Chianese et al. [24] on KISS1 expression. Additionally, KISS1R was also located on Sertoli cells [43]. Sertoli cells are the only type of somatic cells interacting directly with germ cells in the, S.T. They have a major role in regulation of self-renewal, proliferation, and survival of sperm cells [44].

In summary, there is substantial evidence from multiple species to conclude that there is peripheral KISS1/KISS1R localised expression within the testes. However, there is variability in the cell types observed to express kisspeptin or its receptor (see Table 1 and Figure 1). Some of these differences observed may be due to variation between species, age differences within the species and the different experimental methods employed [45].

## 4. Regulation of Testicular Kisspeptin Signaling

*Humoral factors:* The gonads are the sites of synthesis and binding of peptides other than kisspeptin, including GnRH and GnIH [55]. The functional significance of these other neuropeptides in the gonads has been studied in isolation but mostly during puberty [56]. GnRH is a central modulator of testis physiology with paracrine Leydig–Sertoli, Sertoli–germ cell, Sertoli–peritubular cell communications [57,58,59,60]. However, emerging evidence suggests that testicular kisspeptin may have cross-communication with GnRH and GnIH in an autocrine or paracrine manner for regulating gonadal development and function [19].

In men, oestradiol (E2) is primarily synthesised in the testes, which express specific oestradiol receptors, ERa and ERb. An interaction between oestradiol and kisspeptin signaling has been observed in frog testes. In vitro incubation of frog testes with oestradiol (17B-estradiol) induced the expression of KISS1R, the effects of which were attenuated with an oestrogen receptor antagonist ICI182-780. In turn, kisspeptin, kp10, at varying doses, induced a dose-dependent expression of oestradiol receptors, ERa and ERb [24]. Furthermore, in vitro incubation with kp10 (dose 10^−6^M) increased expression of testicular KISS1R verses incubation with the KISS1R antagonist, p234. Lower doses of kp10 ranging from 10^−9^ to 10^−7^ M had no effect on KISS1R expression at 1-hr. However, 4-hr incubation of frog testes with all doses of kp10 (10^−6^ to10^−9^ M) increased the KISS1R expression in a dose dependent manner. This study highlights both dose- and time-dependent regulatory effects of kp10 on expression of KISS1R and oestrogen receptors [24].

There is also emerging evidence that LH has an in vivo and in vitro role in local modulation of KISS1 expression and signaling [21]. Primary Leydig cell culture isolated from the mice testes was incubated with ovine-LH; increases in KISS1 mRNA levels from 3 hr to 18 hr were observed post incubation. Furthermore, Western blotting confirmed an increase in translated kisspeptin protein in adult mice testes at 6 hr post LH incubation. In addition, Salehi et al. [21] observed a single subcutaneous (s/c) injection of GnRH agonist and intraperitoneal (ip) human chorionic gonadotropin (hCG) injection increased LH levels (with no change in FSH) in mice. This was associated with significant increase in testicular KISS1 mRNA and protein expression within 2 hr of treatment compared with saline administration. Conversely, KISS1 expression and protein levels were decreased post ip testosterone via reduction in LH and FSH by negative feedback. This highlights the interaction of various neuropeptides at testicular level with a proposed role of LH in upregulating Leydig cell KISS1 expression and local kisspeptin production. 

*Stage of development:* The reproductive system undergoes multiple changes throughout the postnatal period, from the neonatal period to puberty onset, adulthood, and finally old age [61]. Anjum et al. [19] reported a significant correlation between changes in expression of kisspeptin with GnRH and GnIH in the testis of Parkes strain mice from birth to reproductive senescence. KISS1 expression, analysed by immunostaining of mice Leydig cells, was observed to significantly decrease from birth (day 1) to pre-pubertal testis (4 weeks) with an increase in KISS1 expression during the pubertal period (6 weeks). There was a subsequent decrease in expression of KISS1 in reproductively active mice (15 weeks) with a successive increase during senescence (65 weeks). Similar changes across the lifespan were noted in the GnRH-R (GnRH receptor) expression. Particularly, the increased immunostainings of GnRH, GnIH, kisspeptin, and GnRH-R in Leydig cells during the pubertal period paralleled with the increase in testicular activity reflected by increased levels of cholesterol side-chain cleavage (CYP11A1; steroidogenic enzyme), testosterone and testicular weight of mice. This suggests that these neuropeptides in testicular steroidogenesis and/or sperm function may have regulatory roles at specific stages of reproductive growth. A limitation to Anjum et al.’s study [19] was that expression of KISS1R was not evaluated. However, Wang et al. [42] reported expression of KISS1R mRNA in the testes of 2-, 5-, and 15-week-old mice. This result was confirmed with RT-PCR with no change in KISS1R expression in 2 to 15-week old mice. However, in contrast, the expression of KISS1 and Esr1 (oestrogen receptor) was found to increase during 2–4 weeks of age to attain its highest level at puberty and maintained that expression level until the age of 15 weeks. Expression of genes required for steroidogenesis including luteinizing hormone choriogonadotropin receptor (LHCGR), steroidogenic acute regulatory protein (StAR) and CYP11A1 showed steady increase, with the highest levels in adult mice (at 15 weeks). Therefore, Wang et al. [42] proposed that KISS1 expression in Leydig cells may be correlated with the maturation of Leydig cells during development. Similarly, Salehi et al. [21] reported nil kisspeptin mRNA at post-natal day 7 with an increase in intratesticular KISS1 mRNA levels during mouse development with the highest levels seen at puberty-onset (day 28). This is important in mice as spermatogenesis in mice starts postnatally after 3 weeks. This is perhaps similar to the KISS1 gene upregulation pre-ovulation LH surge seen in rats [62,63]. Additionally, serum LH increased whilst serum kisspeptin reduced one week post gonadectomy indicative of testicular kisspeptin secreted into the serum. However, the authors [21] did not measure expression of KISS1/KISS1R post gonadectomy to support this hypothesis. 

Studies have been carried out on seasonal breeding species to define regional and seasonal-specific patterns of kisspeptin expression [24,64,65]. An increase in pituitary and testicular KISS1R mRNA expression was observed at the end of the winter stasis (February) and reached high levels during the breeding season (April) in frogs in Italy [24]. These studies are summarised in Table 1.

## 5. Effects of Kisspeptin on Sperm Function

Studies using knockout mice, KISS1R antagonist (p234) [66], gonadectomy or chronic exposure to kisspeptin to desensitize KISS1R signaling [67] have contributed to our better understanding of the physiological role of kisspeptin beyond the brain. 

*Humans:* Localisation of KISS1 and KISS1R in mature human spermatozoa suggests a role in regulation of male fertility directly at the level of the male gamete [16]. Kisspeptin (kp13) at a dose of 1μM–10μM triggered a gradual increase in intracellular calcium (Ca^2+^) in spermatozoa, reaching a plateau in 3–6 min. Furthermore, kp13 at higher dose of 10 μM increased sperm motility and transient sperm hyperactivation. However, there were differences in maximal response time to kp13 with approximately 30% of sperm preparations not responding to kp13 at all. The effects of kp13 on sperm motility and hyperactivation were blocked by KISS1R antagonist, p234, suggestive of a direct role of kisspeptin in human spermatozoa. Interestingly, no effect of kp13 on acrosome reaction was observed. 

A pilot study [47] evaluated serum kisspeptin levels in infertile men with sperm parameters defined by WHO criteria [68]. They observed that normozoospermic men had significantly higher serum kisspeptin levels compared to oligoteratozoospermic men who had the least observed serum kisspeptin levels. Moreover, a recent health survey [46] of 666 Chinese student volunteers measured seminal and serum kisspeptin levels and correlated them to semen quality, adjusting for age, BMI, smoking and abstinence time. Median kisspeptin levels were 60,000 times higher in seminal plasma than blood plasma, but no significant association was observed between seminal and serum kisspeptin. However, a positive association between total seminal plasma kisspeptin and three sperm parameters (sperm concentration, total sperm count and total motile count) was observed. Furthermore, there was no paternity information provided and only one semen sample per volunteer was obtained. 

*Rodents:* Similar to human spermatozoa [16], kp10 (doses of 25, 50, 100 μM) triggered a dose-dependent increase in intracellular Ca^2+^ within 5–10 min of exposure, in adult mice spermatozoa [20]. In addition, in vitro fertilisation (IVF) rates, determined by counting the number of two-cell embryos 24 hr after incubation of oocytes with spermatozoa, reduced after treatment of spermatozoa with KISS1R antagonist, p234. Upon entry into the female tract, sperm cells undergo rapid metabolic changes, collectively termed ‘capacitation’, which prepare the sperm cells to reach and fertilise the oocyte. No negative effects on IVF rates were noted when spermatozoa were already fully capacitated proposing a role of kisspeptin in capacitation. In addition, localisation of KISS1 expression in Leydig cells, oviductal tissue and the epididymis epithelium with KISS1R expression in acrosome area of spermatids suggests possible KISS1/KISS1R signaling in sperm maturation, capacitation, acrosome reaction, and/or fertilisation. However, it raises multiple unanswered questions: Does kisspeptin release from epididymis, as part of seminal fluid, assist with maturation of sperm? Does kisspeptin released from Leydig cells have an autocrine or paracrine role in spermatids? Does KISS1R translocate from the acrosome area of spermatids to the plasma membrane during late spermiogenesis or maturation. Different kisspeptin peptides (kp13 versus kp10) with varying doses were used in humans versus rodent studies, therefore the effective kp concentration to elevate intracellular calcium maybe species and cell specific. 

Localisation of KISS1/KISS1R system in germ cells postulates its role in germ cell differentiation. Kp10, in a dose-dependent manner, positively stimulated, proliferative (ID4 and MVH) and differentiative markers (SCP3 and c-Kit) in spermatogonial cells from adult azoospermic and new-born mice cocultured with somatic cells [48]. Additionally, the effect of kisspeptin administration on accessory gland structure, seminal vesicle and prostate have also been documented [69]. Fructose is released from seminal vesicles, and its levels are reflective of seminal vesical function, with fructose metabolic pathways observed in mice spermatozoa. Chronic administration of ip kp10 at different dosage concentrations (1μg, 1 ng, and 10 pg) significantly reduced fructose levels in adult male mice [70].

*Frogs:* KISS1R mRNA was detected in primary and secondary spermatogonial cells by in situ hybridisation at the end of the winter stasis (February) in anuran amphibian, Rana esculenta [24]. With the presence of KISS1R in proliferating spermatogonial cells, the authors concluded that testicular KISS1R regulates the onset of spermatogenic waves. Spermiation is described as the process by which mature spermatids are released from Sertoli cells into the ST lumen prior to their passage to the epididymis [71]. A possible role of kp10 in spermiation in anuran amphibian was investigated with ex vivo treatment of testis with increasing doses of kp10 (10^−9^ to 10^−6^M) with increased number of ST with detached spermatozoa [43]. These effects were blocked by the KISS1R antagonist, p-234. The presence of KISS1R expression in Sertoli cells and peritubular myoid cells suggests a potential role of kp10 in reshuffling of junctional proteins between Sertoli cells and sperm cells, with postulated involvement in sperm release [43].

*Fish:* In pre-pubertal male chub mackerel (Scomber japonicus), in vivo administration of s/c injection of kisspeptin 1–15 peptides over 6 weeks increased gonadosomatic index (GSI (%) = gonad mass/body mass without gonads multiplied by 100), and accelerated spermatogenesis with histological increase in the number of spermatozoa, spermatocytes, and spermatids, whilst the control group only had spermatogonial cells in the testes [26]. Sex steroids are essential for spermatogenesis however this study observed no difference in circulating levels of testosterone post treatment with kisspeptin [25].

In summary, local and direct testicular effects of kisspeptin are summarised in Table 1. These include acceleration of germ cell progression in fish [26], increased spermiation [43]. and alteration of oestradiol signaling in amphibians [24], advancement of germ cell proliferation [48]. in mice, to modulation of intracellular Ca^2+^ in mice and humans [16,20]. Overall, evidence supports that testicular kisspeptin may not be essential for spermatogenesis, but is an important regulator, as male patients with KISS1R mutations respond to exogenous hormonal treatment and successfully achieve fertility, whilst KISS1/KISS1R mutant mice still show low levels of spermatogenesis on a phytoestrogen diet [72].

## 6. Effects of Kisspeptin on Testicular Steroidogenesis: 

Leydig cells synthesise testosterone under LH regulation, in the interstitial compartment of the testes. Steroidogenic genes, such as steroidogenic acute regulatory protein (StAR), cytochrome P450 cholesterol side-chain cleavage (CYP11A1) enzyme and 3-b-hydroxysteroid dehydrogenase (HSD3B1) play critical roles in hormone-regulated steroidogenesis [73].

Several studies have evaluated the use of exogenous kisspeptin on testosterone production to investigate a physiological or potential pharmacological role for peripheral kisspeptin (see Table 1 and Figure 1). Wang et al. [42] concluded that kisspeptin does not affect steroidogenesis in Leydig cells despite their presence of KISS1R expression. Co-treatment of kp10 (1 μM, 5 μM, or 10 μM) with ovine-LH in primary Leydig cell culture of adult mice had no effect on testosterone production. Similarly, neither the activity of CYP11A1 nor HSD3B1, both key steroidogenic enzymes, was altered by kp10.

Mei et al. [12] reported that kp10 (1 μM) does not stimulate in vitro testosterone production from primary mice testes explants. In addition, an immortalized Leydig cell line, MA-10, was used which, similar to Leydig cells, expresses LH receptors and responds to hCG stimulation. There was no progesterone response from administration of kp10 to MA-10 cell line, with or without hCG treatment, despite high concentrations of kp10 (20 μM), refuting any synergistic effects on steroidogenesis. Furthermore, another supplementary study [20] had concordant results whereby primary mice Leydig cells treated with kp10 or kisspeptin receptor antagonist with or without ovine LH did not affect testosterone secretion.

Interestingly, peripheral intravenous (IV) administration of kisspeptin receptor antagonist, p234 had no effect on mean plasma testosterone levels at 360 min in adult male rhesus monkeys [74]. Along the same lines, in vitro testicular tissue of adult male rhesus monkeys, incubated with kisspeptin at multiple doses (1, 10, 100, 1000 pM) did not affect testosterone or inhibin secretion at 2 hr refuting any direct role of kisspeptin on the secretion of these hormones [52]. In summary, these few studies suggest that kisspeptin may not directly interact with testicular tissue in the regulation of testosterone. 

In contrast, several other studies have investigated the direct testicular effects of chronic versus acute exposure to kisspeptin. Continuous s/c (50 nmol/day 6–36 hr) infusion of kp54 led to testicular seminiferous tubule degeneration and apoptosis in male Wistar adult rats [50]. Individual seminiferous tubules showed varying amounts of degeneration–variable cell maturation arrest, generation of multi-nucleated spermatid giant cells, sloughing and apoptosis of germ cells, with complete atrophy of germ cells. These changes were visible as early as 12 hr post infusion, whilst the LH/FSH levels were still raised, suggestive of degeneration pre-desensitisation of HPG axis. Interestingly, neither Leydig cell morphology nor any other tissues (epididymis, vas deferens or prostate) underwent degenerative changes. Inhibin B halved following two days of chronic s/c kp54 infusion. As inhibin B is predominantly produced by Sertoli cells [75], this is suggestive of Sertoli cell degeneration and potential disruption of spermatogenesis. Similarly, single acute doses (5/50 nmol) of peripheral kp54 s/c injection led to a dose-dependent testicular degeneration. However, pre-treatment with cetrorelix, a GnRH antagonist, blocked the kp-induced testicular degeneration and hormone level changes suggesting that the degeneration is likely to be centrally-mediated. The authors postulated that the ‘LH surge’ post kp54 administration could have led to over-excitation of the HPG axis with local release of proinflammatory mediators leading to testis inflammation, hypoxia and degeneration. Similarly, chronic ip injection of kp10 (1 ng or 1 mg) twice per day for 12 consecutive days into a maturing 35-day-old rat testes reduced LH and testosterone levels, with no effect on FSH levels [49]. Furthermore, it also led to a reduction in sperm maturation and the degeneration of the seminiferous tubules. Similar degenerative effects of continuous administration of kp-10 has also been reported in rat seminal vesicles and pre-pubertal prostate gland [76]. Other studies have supported these observed negative findings. Administration of kp54 (50 nmol/day) for 13 days using mini-osmotic pumps implanted subcutaneously in the interscapular region of male rats significantly decreased serum hormone levels (LH, FSH, and testosterone levels), testis weight, sperm count and motility through direct desensitization of the HPG axis [51].

Continuous IV infusion of kisspeptin 45–54 over 4 days, in adult rhesus monkeys, maintained plasma testosterone levels even when LH levels fell [53]. The testosterone:LH ratio was high, for any given level of, L.H.; in the treatment group compared to the control, suggesting a possible intra-testicular action of kisspeptin contributing to the amplified testosterone levels. 

Another important in vivo study [54] proposed that IV kp10 administration (50 μM) significantly increased hCG-stimulated testosterone levels in pituitary-clamped (acyline-treated) monkeys compared to hCG + saline, suggesting kisspeptin augments the LH response in Leydig cells. Acyline is a GnRH receptor antagonist used to assess the peripheral action of kisspeptin independent of the central LH action.

In summary, fewer studies have demonstrated the potentiating effect of peripheral kisspeptin on steroidogenesis, whilst many others have been unable to reproduce these findings, with multiple reports of negative or null effects from chronic peripheral infusion of kisspeptin. It is difficult to conclude assertively with conflicting results. Moreover, different studies used different kp-derivatives at varying doses with different pharmacokinetic outlines, routes and duration of administration. Experimental methods have also differed between studies. There are also clear inter-species differences, as well as differences in age of the animals within the same species, which may also lead to variable findings. 

## 7. Conclusions

There is clear evidence for the distribution and expression of KISS1 and KISS1R in the testes, as well as direct biological effects of kisspeptin in the testis. Yet, the patterns of testicular expression and physiological autocrine and paracrine characteristics remain inconclusive. Furthermore, several aspects of testicular function are different between mammals and non-mammalian species. Although tissue and cell culture experiments show that kisspeptin may act on testicular germ and somatic cells, its direct effect in in vivo systems has not yet been fully elucidated. Further understanding of the role of kisspeptin in testicular function could provide a successful tool for future development of new potential therapeutic targets in the treatment of male fertility disorders. Careful consideration of dose, method of administration, and isoforms of kisspeptin are likely to concentrate extensive research efforts in the near future. 

## Figures and Tables

**Table 1:** Distribution, Regulation and Effects of KISS1/KISS1R on Testicular Function in Various Species.

Species	Distribution of KISS1 & KISSR	Regulation	Effects on Sperm Function	Effects on Testicular Steroidogenesis
Human	KISS1 & KISS1R: head (post-acrosomal region), neck, and flagellum of spermatozoa [16]. KISS1: seminal plasma [46].		kp13: ▲ intracellular Ca^2+^ in spermatozoa with ▲ sperm motility and transient sperm hyperactivation [16]. ▲ serum kp levels in normozoospermic vs. oligotetatozoospermic men [47].	
Mice	KISS1 & KISS1R: Leydig cells [12,19,20,21,42], round [12], and elongated spermatids [20]. KISS1: primordial germ cells [19], and epididymal epithelium [20].	LH: ▲ KISS1 mRNA [21]. T: ▼ KISS1 expression and protein [21]. GnRH, GnIH and GnRH-R: autocrine or paracrine modulation of KISS1 [19].	kp10: ▲intracellular Ca^2+^ in spermatozoa [20], ▲proliferation and differentiation markers on spermatogonial cells [48]. kp: role in sperm capacitation [20].	kp10: addition of LH had no effect on T production [20,42] or steroidogenic enzymes [42]. kp10: no effect on progesterone levels ± hCG [12]. Chronic kp10 injection ▼ LH and T levels [49]. kp54: continuous infusion ▼ inhibin B [50], LH, FSH and T levels [51] and seminiferous tubule degeneration [50].
Monkey	KISS1: Leydig cells [18], spermatocytes and spermatids [17]. KISS1R: Sertoli cells and spermatocytes [17,18].			kp: no effect on T or inhibin secretion [52]; Continuous kp infusion maintains T levels [53]. kp10: ▲ hCG-stimulated T levels [54].
Frog	KISS1R: Sertoli cells, primary & secondary spermatogonial cells, primary spermatocytes, and peritubular myoid cells [24].	E2: ▲KISS1R. kp10: ▲ KISS1R [24].	kp10: ▲ number of seminiferous tubules with detached spermatozoa [43].	

GnRH, gonadotrophin-releasing hormone; GnIH, gonadotrophin inhibiting hormone; GnRH-R, gonadotrophin releasing hormone receptor; hCG, human chorionic gonadotropin;, L.H.; Luteinising hormone; FSH, follicle stimulating hormone; E2, oestradiol;, E.R.; oestrogen receptor; kp, kisspeptin; Ca^2+^, calcium;, T.; Testosterone. ▲: increase(d), ▼: decrease(d).

## Abbreviations

GnRH	gonadotrophin-releasing hormone
LH	luteinising hormone
FSH	follicle-stimulating hormone
ST	seminiferous tubules
HPG	hypothalamic-pituitary-gonadal axis
NKB	Neurokinin B
GnIH	Gonadotrophin inhibiting hormone
RT-PCR	Reverse transcriptase-polymerase chain reaction
ARC	arcuate nucleus
E2	oestradiol
kp	kisspeptin
s/c	subcutaneous
ip	intraperitoneal
hCG	human chorionic gonadotropin
GnRH-R	GnRH receptor
CYP11A1	cytochrome P450 cholesterol side-chain cleavage
Esr1	oestrogen receptor
LHCGR	luteinizing hormone choriogonadotropin receptor
StAR	steroidogenic acute regulatory protein
Ca^2+^	calcium
IVF	in vitro fertilisation
GSI	gonadosomatic index
HSD3B1	3-bhydroxysteroid dehydrogenase
IV	intravenous
